# De novo comparative transcriptome analysis of a rare cicada, with identification of candidate genes related to adaptation to a novel host plant and drier habitats

**DOI:** 10.1186/s12864-019-5547-y

**Published:** 2019-03-07

**Authors:** Zehai Hou, Cong Wei

**Affiliations:** State Key Laboratory of Crop Stress Biology for Arid Areas, and Key Laboratory of Plant Protection Resources and Pest Management, Ministry of Education, College of Plant Protection, Northwest A&F University, Yangling, 712100 Shaanxi China

**Keywords:** Cicadidae, Midgut, Host shift, Host adaptation, Transcriptional variation, Plasticity, RNA-Seq

## Abstract

**Background:**

Although the importance of host plant chemistry in plant–insect interactions is widely recognized, our understanding about the genetic basis underlying the relationship between changes in midgut proteins and adaptation of plant-feeding insects to novel host plants and habitats is very limited. To address this knowledge gap, the transcriptional profiles of midguts among three populations of the cicada *Subpsaltria yangi* Chen were compared. Among which, the Hancheng (HC) and Fengxiang (FX) populations occurring in the Loess Plateau feed on *Ziziphus jujuba* Mill. var. *spinosa* (Bunge) Hu ex H. F. Chow, while the population occurring in a much drier habitat in the Helan (HL) Mountains is locally specialized on a chemically divergent plant, *Ephedra lepidosperma* C. Y. Cheng.

**Results:**

Based on comparative analysis, 1826 (HL vs HC) differentially expressed genes (DEGs) and 723 DEGs (HL vs FX) were identified between the populations utilizing different host plants, including 20, 36, 2, 5 and 2 genes related to digestion, detoxification, oxidation-reduction, stress response and water-deprivation response, respectively, and 35 genes presumably associated with osmoregulation. However, only 183 DEGs were identified between the HC and FX populations, including two genes related to detoxification, two genes related to stress response, and one gene presumably associated with osmoregulation. These results suggest that the weakest expression differences were between the populations utilizing the same host plant and occurring in the closest habitats, which may help explain the metabolic mechanism of adaptation in *S. yangi* populations to novel host plants and new niches.

**Conclusions:**

The observed differences in gene expression among *S. yangi* populations are consistent with the hypothesis that the host plant shift and habitat adaptation in the HL population was facilitated by differential regulation of genes related to digestion, detoxification, oxidation-reduction, stress response, water-deprivation response and osmoregulation. The results may inform future studies on the molecular mechanisms underlying the relationship between changes in midgut proteins and adaptation of herbivorous insects to novel host plants and new niches.

**Electronic supplementary material:**

The online version of this article (10.1186/s12864-019-5547-y) contains supplementary material, which is available to authorized users.

## Background

For herbivorous insects, incorporation of a novel host into the diet and subsequent formation of distinct host associations play an important role in the early step of speciation process [1]. Plants produce a wide range of allelochemicals to defend against herbivore attack while, in turn, insects have evolved different ways of coping with the chemical barriers. Due to the remarkable diversity of plant secondary compounds, an insect is inevitably confronted with different chemical environments when undergoing a host switch [2]. Consequently, host shift is an important driver of phenotypic and genetic changes for herbivorous insects [3]. Although the importance of insect adaptation to host chemistry has long been recognized, our understanding of the genetic basis of adaptation to new hosts is still insufficient [2].

**Fig. 1:**
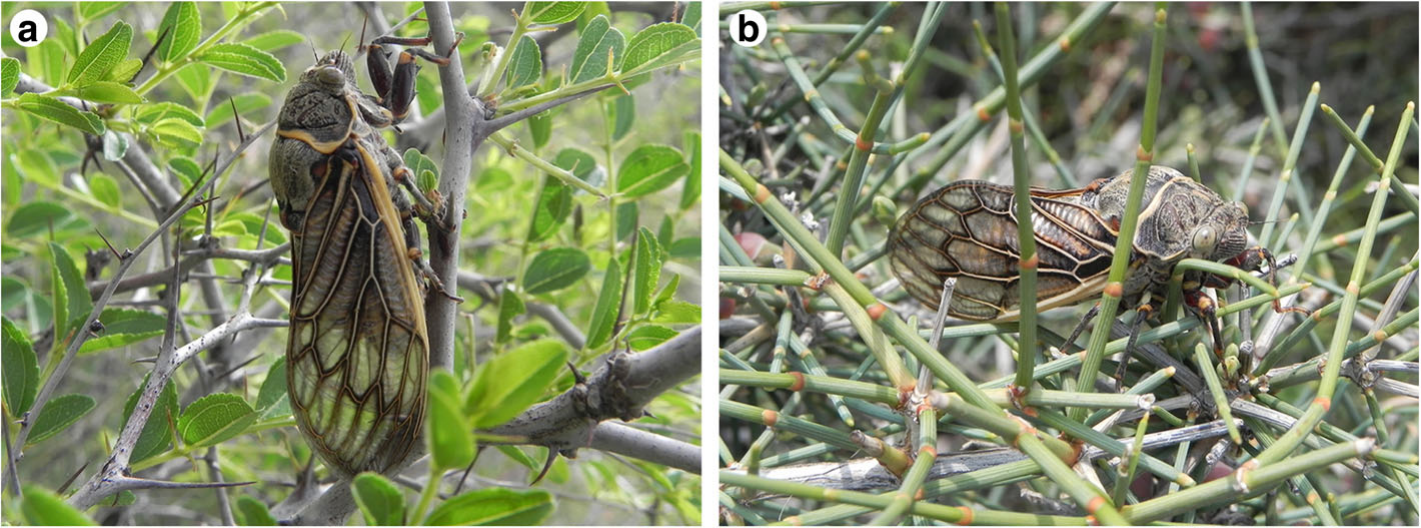
Host plants of *Subpsaltria yangi*. **a** A *S. yangi* feeding on *Ziziphus jujuba* var. *spinosa.* **b** A *S. yangi* feeding on *Ephedra lepidosperma.* Both taken by Z. Hou

*Subpsaltria yangi* Chen (Cicadidae: Tibicininae) is an endemic, Chinese cicada species [4–6], and male’s intraspecific sexual mimicry in this species was revealed recently [7]. This rare species had been known to be distributed in the Loess Plateau and adjacent areas in Shaanxi Province and the Helan (hereafter HL) Mountains, Ningxia Hui Nationality Autonomous Region [4, 5, 7]. During the field investigations of *S. yangi* since 2011, a few more populations were discovered from the Loess Plateau and adjacent areas in Shaanxi, Shanxi and Gansu provinces [8]. We found that most populations of *S. yangi* occurring in the Loess Plateau and adjacent areas specialize on the sour jujube *Ziziphus jujuba* Mill. var. *spinosa* (Bunge) Hu ex H. F. Chow (Rhamnaceae) (Fig. 1a). However, the population occurring in the Helan Mountains, surrounded by deserts or semi-deserts, utilizes *Ephedra lepidosperma* C. Y. Cheng (Ephedraceae) (Fig. 1b), although sympatric *Z. jujuba* var. *spinosa* is available (see Results). Genetic evidence and acoustic analysis of the calling song of males significantly indicate that the HL population is genetically differentiated from other populations of *S. yangi*, which provide a possible example of incipient speciation in insects [8]. This, coupled with the fact that *Z. jujuba* var. *spinosa* are abundant but not utilized as main host in the Helan Mountains, suggests that the population from the Helan Mountains has experienced a ‘host shift’.

Identifying transcriptional changes associated with host shifts is an important step in understanding the molecular mechanisms of herbivorous insects adaptating to their new hosts [2, 9, 10]. Previous studies have demonstrated transcriptional regulations associated with insects feeding on different hosts. For example, research on host shift in a cactophilic fly, *Drosophila mojavensis*, has identified cytochrome P450s, glutathione S-transferases and UDP-glycosyltransferases as important gene classes likely involved in cactus utilization [11]. Research on host shift in another cactophilic fly, *D. mettleri*, revealed that host shift induces a number of genes involved in processes relevant to host adaptation, e.g., detoxification, central metabolism and chemosensory pathways [9]. De Panis et al. [12] suggested that transcriptome modulation during host shift is driven by secondary metabolites in the desert cactophilic fly *D. buzzatii*. Recently, Zhong et al. [10] suggested that transcriptional regulation of genes related to digestion, detoxifcation and ribosome may play an important role in adaptation of the striped stem borer *Chilo suppressalis* to a new host, the water-oat *Zizania latifolia*.

Cicadas are root pests feeding on the xylem fluids of their host plants and then emerge from the soil to use the tree canopies for mating, oviposition, and also additional feeding [13, 14]. Populations of *S. yangi* feeding on different host plants may confront different chemical environments. The plant *Z. jujuba* var. *spinosa* is a thorny, deciduous plant widely distributed throughout the tropical and subtropical regions of the world [15]. The major compounds of branches and leaves of *Z. jujuba* var. *spinosa* are rutin, catechin, lupeol, quercetin-3-O-β-D-glucoside, zizyberanalic, epiceanothic and betulic acid [16, 17]. In contrast, plants of the genus *Ephedra* are known to contain six types of alkaloids, i.e., ephedrine, pseudoephedrine, methylephedrine, methylpseudoephedrine, norephedrine and norpseudoephedrine, which have been used in traditional Chinese medicine for at least 5000 years [18]. Adult cicadas feed exclusively on the xylem fluid from branches of their host plants. Cicada nymphs are long lived underground and feed exclusively on the xylem sap from roots of their host plants. The cicada *S. yangi* may be a serious pest on the medicinal plant *E. lepidosperma.* Although the chemical composition in *E. lepidosperma* is not documented detailedly, this plant has been reported containing two types of alkaloids, ephedrine and pseudoephedrine [19]. Previous studies have shown that breeding in an alkaloid-rich host plant *Trichocereus terscheckii* [20, 21] affects fitness in *D. buzzatii*, such as decreasing viability, body size and starvation resistance, and extending development [22–25]. Therefore, adaptation to a new host plant such as *E. lepidosperma* for *S. yangi* entails the acquisition of mechanisms aimed to face such chemical challenges. This, coupled with geographic variation in host plant specialization, makes the *S. yangi* system especially amenable for studies on the genetic basis of new host adaptation.

Herbivorous insects show variability in their ability to use plants, which is based on differences in preference, ingestion speed, digestion efficiency, and detoxification [26, 27]. Olfaction in antennae is essential for regulating insect behaviors such as host preference and oviposition site selection [28]. Many olfactory-related proteins are responsible for perceiving plant volatiles and mediating host selection of phytophagous insects [29, 30]. Recently, Qi et al. [28] conducted transcriptome analysis based on RNA-Seq data from antennae of *S. yangi* to better understand the olfactory mechanisms in *S. yangi* which has a very narrow host range far fewer than diets of most other cicadas. They identified a number of olfactory genes of *S. yangi*, which provide direct evidence for future research of the olfactory system of *S. yangi* at the molecular level and provide information for elucidation of the molecular mechanisms and evolution of chemosensation in sap-sucking insects.

The midgut, the second largest organ in the insect body, actively interacts with the physical environment [31], which is the entry site for harmful bacteria, viruses and toxins, as well as for food and water [32]. The midgut also plays critical roles in digestion and nutrient uptake and, in particular, detoxification and oxidative stress responses. However, our understanding about the mechanisms underlying the relationship between changes in midgut proteins and adaptation of plant-feeding insects to different host plants is still very limited. Comparative studies are needed to elucidate mechanisms underlying the relationship between the changes in midgut proteins and the adaptation of insects to novel hosts. Thus, we compared gene expression in the midgut of different populations of *S. yangi* respectively feeding on *Z. jujuba* var. *spinosa* and *E. lepidosperma*. Our main goal was to detect differentially expressed genes (DEGs) in response to different host plants and habitats, and to test the hypothesis that the host plant shift in *S. yangi* is facilitated by differential regulation of genes related to digestion and detoxification. The results may also be helpful to explain the metabolic mechanism in *S. yangi* to adapt to new habitats and mechanisms of speciation in herbivorous insects.

## Results

### Host plant utilization

For the HL population, host plant utilizations of 402 and 345 individuals were recorded during the whole emergence period of 2016 and 2017, respectively. We found significantly more adults feeding on *E. lepidosperma* (*N* = 379) than on *Z. jujuba* var. *spinosa* (*N* = 23) (ratio = 379: 23, χ^2^ = 313.50, *P* < 0.001) in 2016, and the individuals feeding on the former continued to predominate (ratio = 338: 7, χ^2^ = 315.65, *P* < 0.001) in 2017. The samples in 2016 and 2017 were pooled to obtain a significantly biased ratio toward to *E. lepidosperma* (ratio = 717: 30, χ^2^ = 629.98, *P* < 0.001). In contrast, our field investigation confirmed that the populations occurring in Hancheng (hereafter HC) and Fengxiang (hereafter FX) of Shaanxi Province are all feeding on *Z. jujuba* var. *spinosa*.

### Summary of sequences and assembly

To quantify the gene expression patterns of midgut samples, we constructed nine cDNA libraries and then subjected them to deep sequencing using Illumina HiSeq2000. In total, 70.26 Gb high quality sequences were obtained from the transcriptome sequencing of midgut samples, ranging from 6.34 to 10.59 Gb per sample. The average error rates of the sequences are 0.01–0.02%, and more than 91% of the bases with error rates < 0.1% (Additional file 1: Table S1). The sequencing data were assembled into 418,777 transcripts with length ranging from 201 to 33,432 bases (mean length = 770 bases, and median length = 368 bases). As a result, 234,592 unigenes were obtained (mean length = 1149 bases, and median length = 653 bases). This assembly had a high degree of completeness with a BUSCO score of 96.4%, of which 693 (41.8%) were complete single-copy BUSCOs, 906 (54.6%) were complete duplicated BUSCOs, 27 (1.6%) were fragmented BUSCOs, and 32 (1.9%) were missing BUSCO orthologs out of the 1658 BUSCO groups searched.

### Functional annotation of the midgut unigenes

In sum, 89,441 unigenes (38.1% of the total unigenes) were annotated in at least one of the databases used in our study. Those unigenes were mostly annotated in the Nr database (30.9%) (Additional file 1: Table S2). The highest percentage of unigene sequences were matched with *Zootermopsis nevadensis* (22.7%), followed by *Acyrthosiphon pisum* (9.2%), *Lasius niger* (5.7%), *Tribolium castaneum* (4.6%), and *Diaphorina citri* (4.5%) (Additional file 1: Table S3).

Based on the Gene Ontology (GO) analysis, 59,179 (25.2%) unigenes were mapped and clustered into cellular component, biological process and molecular function categories. The top two subcategories were: 1) ‘cellular process’ and ‘metabolic process’ in biological process category, 2) the ‘cell’ and ‘cell part’ in cellular component category, and 3) the ‘binding’ and ‘catalytic activity’ in molecular function category (Additional file 2: Figure S1). The results of Eukaryotic Orthologous Groups (KOG) analysis show that the matched 28,896 unigenes are divided into 26 categories. The dominant category includes ‘general functional prediction only’, ‘signal transduction mechanisms’, and ‘posttranslational modification, protein turnover, chaperones’ (Additional file 3: Figure S2). Kyoto Encyclopedia of Genes and Genomes (KEGG) analysis shows that the matched 22,621 (9.6%) unigenes are assigned into 229 pathways, of which the top 32 are depicted in Additional file 4: Figure S3. The most well-represented metabolic pathways are involved in ‘carbohydrate metabolism’ (1383), ‘lipid metabolism’ (1133), ‘amino acid metabolism’ (958), respectively (Additional file 4: Figure S3).

### Identification of SSRs

**Fig. 2:**
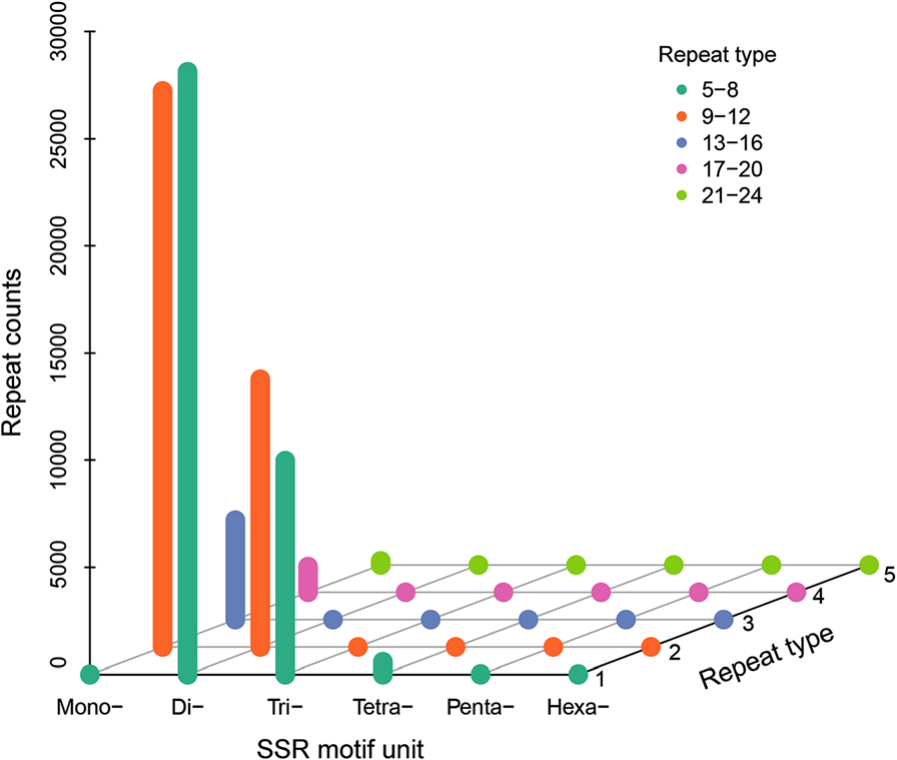
Distribution of SSR motifs. The different color bars represent different repeat types (repeat number ranges of SSR motif unit)

We detected 83,176 SSRs, and the most abundant type of SSR motifs are dinucleotide repeats mainly of (TG/GT/AC/CA)_n_ (48.8%) (Fig. 2). About 38.5% of the SSRs are mononucleotide repeats mainly of (A/T)_n_. Primers were successfully designed for 44,101 SSRs. For the purpose of molecular marker development, complex SSRs [e.g., (TTC)_7_(TTA)_7_, (CG)_6_catg(CA)_6_] and those with a motif unit size less than two nucleotides were removed. As a result, 18,670 SSR primers were obtained (Additional file 5: Table S4). The SSRs identified in our study may be useful for developing molecular markers to detect the genetic structure among populations of *S. yangi*.

### Differential gene expression analysis

**Fig. 3:**
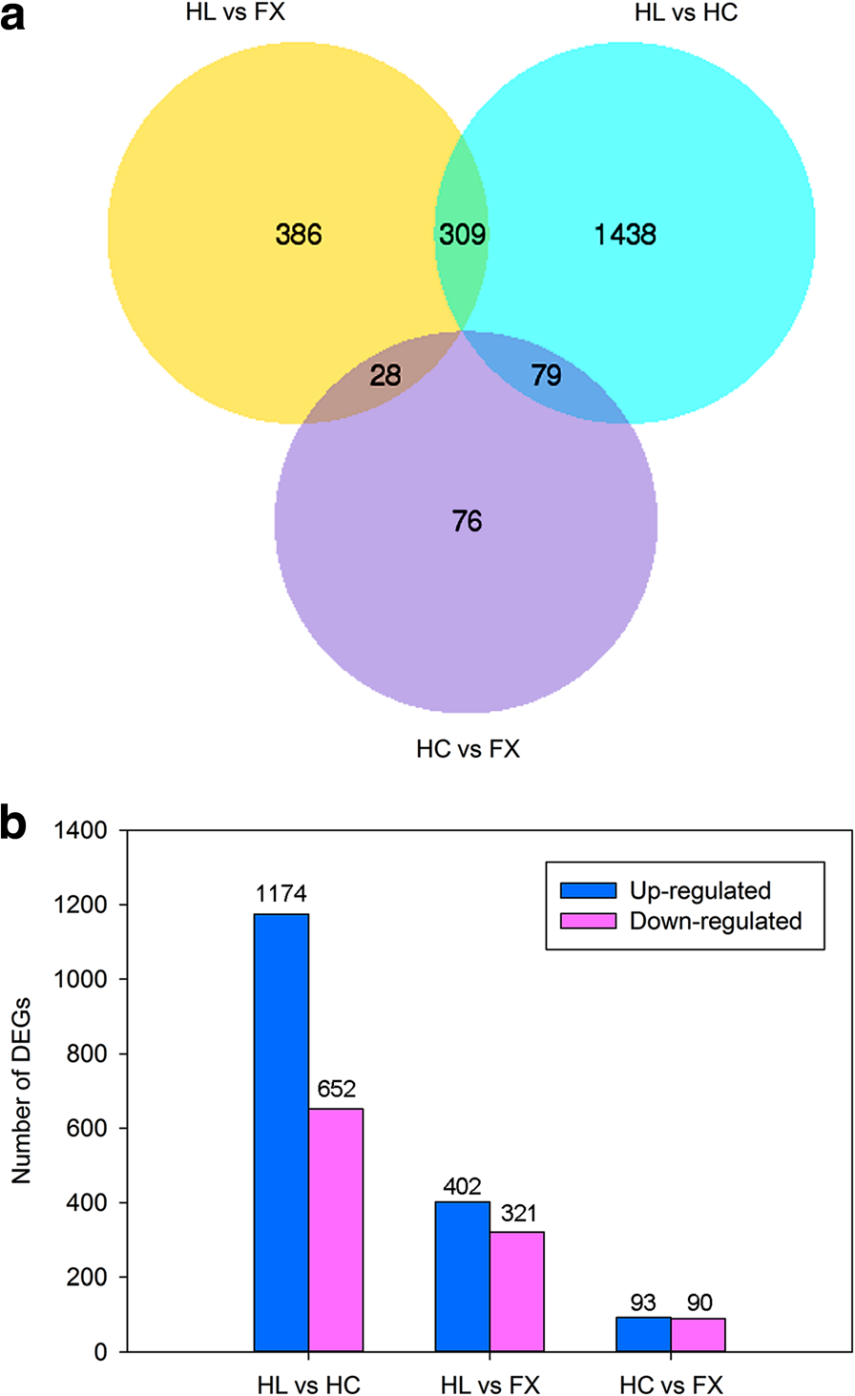
Analysis of DEGs expression among the three populations of *S. yangi*. **a** The number of common and unique unigenes express among the three populations. **b** The number of significantly up- and down-regulated unigenes between two compared populations. FX: the population occurring in Fengxiang; HC: the population occurring in Hancheng; HL: the population occurring in the Helan Mountains

An overall view of gene expressions of midgut of *S. yangi* is presented in the hierarchical clustering heatmap (Additional file 6: Figure S4). The midgut sample of the HC population and that of the FX population cluster together, indicating a relatively high similarity in patterns of gene expression. Venn diagrams were used to summarize the number of differentially expressed genes (DEGs) among the three populations (Fig. 3a). First, we compared the expression profiles between HC (feeding on *Z. jujuba* var. *spinosa*) and FX (feeding on *Z. jujuba* var. *spinosa*), which represent the closest rearing condition in nature. It results in 183 DEGs identified in HC compared with FX, of which 93 were up-regulated and 90 were down-regulated (Fig. 3b; Additional file 7: Table S5). Second, we evaluated the differences in gene expression between the midgut samples of HL (feeding on *E. lepidosperma*) and HC, and detected 1826 DEGs, including 1174 up-regulated genes and 652 down-regulated genes (Fig. 3b; Additional file 8: Table S6). Then we analysed the differences in gene expression between HL and FX, and identified 723 DEGs, of which 402 were up-regulated and 321 were down-regulated (Fig. 3b; Additional file 9: Table S7). There are 309 DEGs shared between the latter two pairwise comparisons (Fig. 3a).

**Fig. 4:**
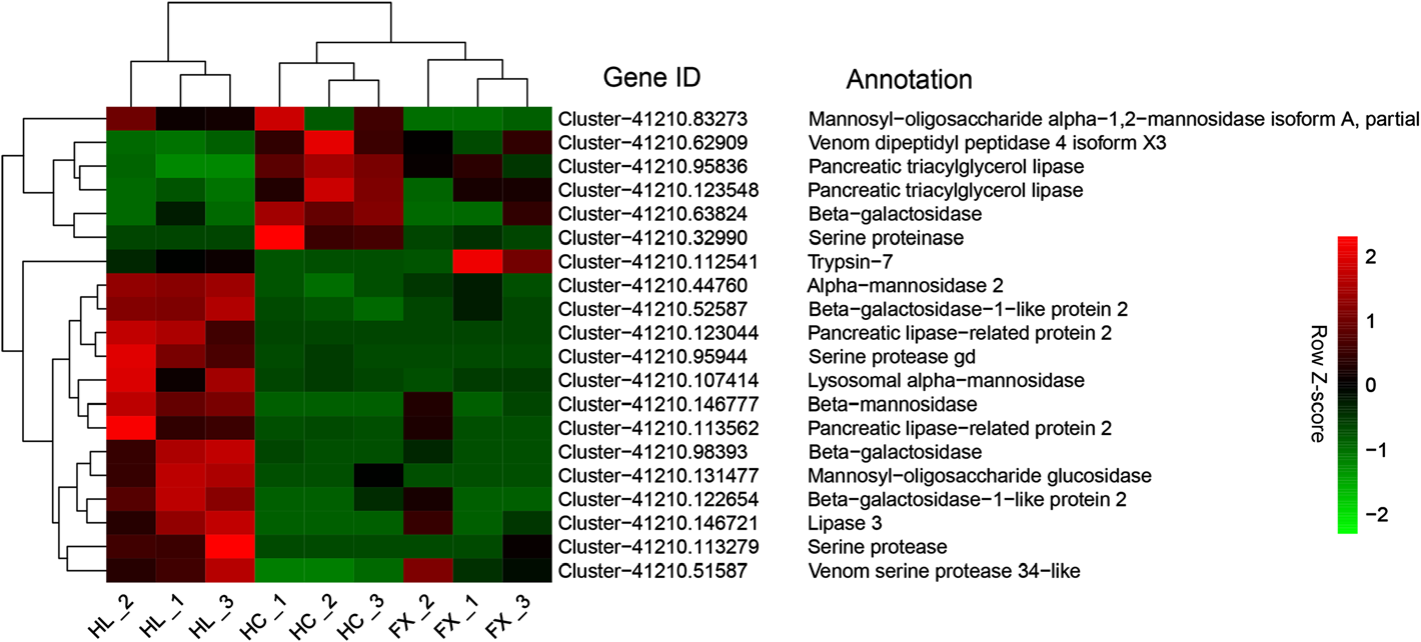
Heatmap of normalized FPKM of DEGs related to digestion. The Z-score represents the deviation from the mean by standard deviation units. Red color indicates up-regulated expression, whereas green color indicates down-regulated expression. FPKM: fragments per kilobase of transcript per million fragments mapped; FX: the population occurring in Fengxiang; HC: the population occurring in Hancheng; HL: the population occurring in the Helan Mountains

**Fig. 5:**
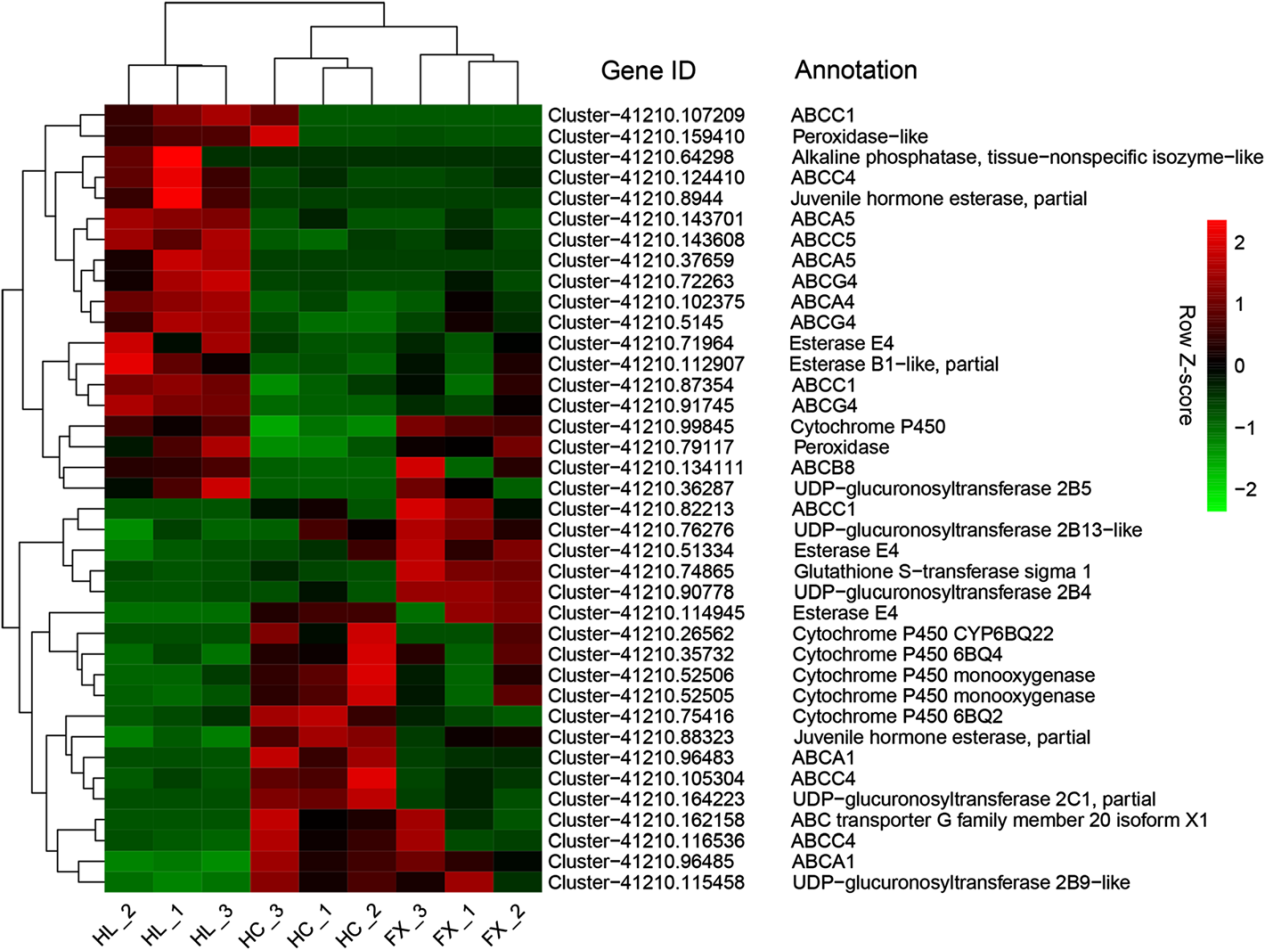
Heatmap of normalized FPKM of DEGs related to detoxification and oxidation-reduction. The Z-score represents the deviation from the mean by standard deviation units. Red color indicates up-regulated expression, whereas green color indicates down-regulated expression. FPKM: fragments per kilobase of transcript per million fragments mapped; FX: the population occurring in Fengxiang; HC: the population occurring in Hancheng; HL: the population occurring in the Helan Mountains

In this study, we mainly focused on the transcriptional changes associated with a host plant shift in *S. yangi*. We identified 20 DEGs related to digestion in the comparative sets ‘HL vs HC’ and ‘HL vs FX’, encoding nine carbohydrases, five lipases and six proteases, and most of them were up-regulated in the HL population when compared with the HC and FX populations (Fig. 4; Additional file 10: Table S8). Thirty-five putative detoxification-related unigenes were also identified, including six cytochrome P450 monooxygenases (P450s), five UDP-glycosyltransferases (UGTs), six carboxylesterases (CEs), one glutathione S-transferase (GST) and 17 ATP-binding cassette (ABC) transporters (Fig. 5; Additional file 11: Table S9). Among which, most P450s and UGTs were down-regulated in the HL population when compared with the HC and FX populations. In addition to the 35 putative detoxification-related unigenes, we also identified an *alkaline phosphatase, tissue-nonspecific isozyme-like* gene (Cluster-41,210.64298) exclusively expressed in the HL population (Fig. 5; Additional file 11: Table S9), indicating this gene may play a significant role in alkaloid resistance when *S. yangi* feeds on *E. lepidosperma*. We also found that two DEGs encoding peroxidase, i.e., one peroxidase (Cluster-41,210.79117) and one peroxidase-like (Cluster-41,210.159410), were up-regulated in the HL population when compared with the HC and FX populations (Fig. 5; Additional file 11: Table S9), which might play a role in protective response to oxidative stress, e.g., reactive oxygen species (ROS) ingested during feeding or food processing.

**Fig. 6:**
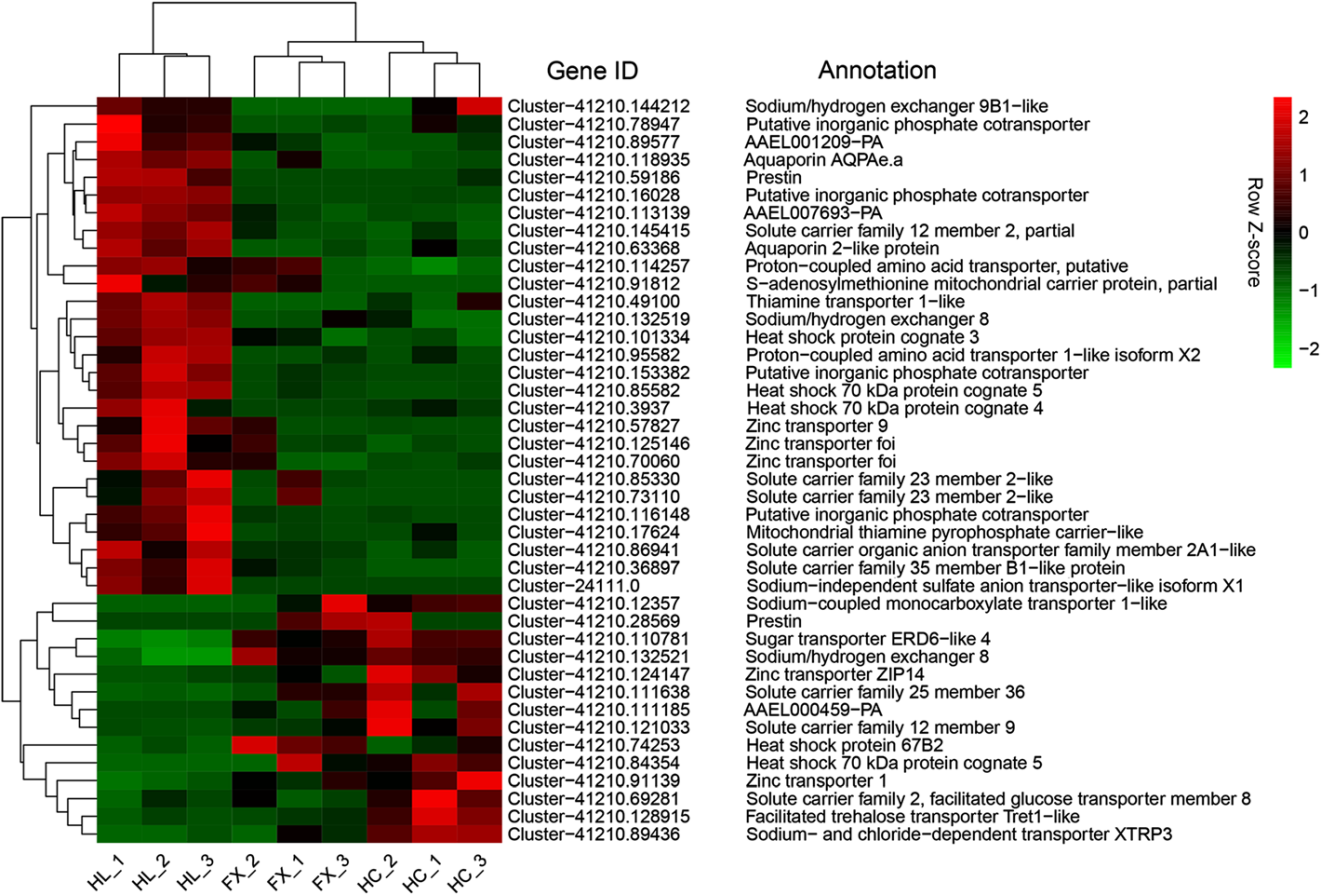
Heatmap of normalized FPKM of DEGs related to stress response, water-deprivation response and putative osmoregulation. The Z-score represents the deviation from the mean by standard deviation units. Red color indicates up-regulated expression, whereas green color indicates down-regulated expression. FPKM: fragments per kilobase of transcript per million fragments mapped; FX: the population occurring in Fengxiang; HC: the population occurring in Hancheng; HL: the population occurring in the Helan Mountains

Besides coping with the host shift from *Z. jujuba* var. *spinosa* to *E. lepidosperma*, the HL population should at the same time be able to adapt to the habitats which are surrounded by deserts or semi-deserts. In our present study, we found that five unigenes encoding heat shock proteins (Hsp) were differentially expressed in the HL population when compared with the HC and FX populations, including one heat shock protein cognate 3, one heat shock 70 kDa protein cognate 4, two heat shock 70 kDa protein cognate 5 and one heat shock protein 67B2 (Fig. 6; Additional file 12: Table S10). We found that two DEGs encoding aquaporins, including one aquaporin AQPAe.a and one aquaporin 2-like protein, were up-regulated in the HL population when compared with the HC and FX populations (Fig. 6; Additional file 12: Table S10), suggesting that they may play an important role in response to water deprivation for the HL population which occurs in the desert or semi*-*desert environments of the Helan Mountains. In addition to Hsps and aquaporins, we also identified 35 DEGs encoding solute carriers (Slc), and majority of them were up-regulated in the HL population when compared with the HC and FX populations (Fig. 6; Additional file 12: Table S10).

However, using the same analytical procedures, we only identified five unigenes, including one *UGT*, one *ABC transporter*, two *Hsps* and one *Slc* genes, differentially expressed in the comparative set ‘HC vs FX’ (Table 1). These differences in gene expression suggest that the weakest expression differences were between the populations utilizing the same host plant and occurring in the closest habitats. These observed differences in gene expression between populations utilizing different host plants and occurring in different habitats may help explain the metabolic mechanism of adaptation in *S. yangi* populations to novel host plants and new niches.

**Table 1:** Differentially expressed genes identified in the comparative set ‘HC vs FX’

Gene ID	Annotation	log_2_FC (HC vs FX)	*q* value
Cluster-41,210.79881	UDP-glucuronosyltransferase 2B4	+∞	2.31E-05
Cluster-36,972.0	ABC transporter G family member 23	–∞	3.25E-02
Cluster-41,210.118981	Heat shock 70 kDa protein cognate 4	+∞	5.39E-04
Cluster-41,210.165451	30 kDa heat shock protein	+∞	3.08E-03
Cluster-41,210.137221	Sodium-dependent phosphate transporter 2	7.58	8.05E-03

*FC* fold change, *FX* the population occurring in Fengxiang, *HC* the population occurring in Hancheng; +∞: genes exclusively expressed in the HC population; −∞: genes exclusively expressed in the FX population

### Mean fixation index values of individual genes

Mean fixation index (Fst) values of individual genes varied broadly in comparisons between populations occurring in different habitats and utilizing different host plants. Mean Fst values of *venom serine protease 34-like* gene and *solute carrier family 12 member 2, partial* gene between HL and FX are 0.08 and 0.11, respectively, indicating a moderate level of genetic differentiation (Table 2). Mean Fst value of *ABCC4* gene between HL and HC is 0.16 (Table 3), also indicating a moderate level of genetic differentiation. While mean Fst values of *heat shock protein cognate 3* gene and *heat shock protein 67B2* gene between HL and FX are 0.47 and 1, respectively (Table 2), indicating a high level of genetic differentiation. Mean Fst values of *venom serine protease 34-like* gene, *cytochrome P450 6BQ4* gene, *solute carrier family 12 member 2, partial* gene, *heat shock protein cognate 3* gene and *heat shock protein 67B2* gene between HL and HC are 0.45, 0.57, 0.63, 0.80 and 0.25, respectively (Table 3), also indicating a high level of genetic differentiation.

**Table 2:** Population genetics and expression data for the genes in the comparative set ‘HL vs FX’

Gene ID	Nr Description	Tajima’s D HL	Tajima’s D FX	Mean Fst	log_2_FC (HL vs FX)	*q* value
Cluster-41,210.51587	Venom serine protease 34-like	1.75	0.31	0.08	0.42	1
Cluster-41,210.75416	Cytochrome P450 6BQ2	0.96	1.97	−0.23	−0.35	1
Cluster-41,210.35732	Cytochrome P450 6BQ4	0	−0.05	5.55E-17	− 1.98	9.98E-01
Cluster-41,210.51334	Esterase E4	NA	0	−0.096	−3.41	1.63E-02
Cluster-41,210.96485	ABCA1	NA	0	−1.14E-16	−2.76	7.54E-02
Cluster-41,210.105304	ABCC4	0	−1.13	−0.12	−1.03	1
Cluster-41,210.91139	Zinc transporter 1	NA	0	−0.096	−3.0	1.31E-01
Cluster-41,210.111638	Solute carrier family 25 member 36	0	0	−0.29	−4.91	2.54E-01
Cluster-41,210.145415	Solute carrier family 12 member 2, partial	NA	−0.19	0.11	2.28	3.10E-01
Cluster-41,210.101334	Heat shock protein cognate 3	−1.13	0	0.47	1.73	9.51E-01
Cluster-41,210.74253	Heat shock protein 67B2	NA	NA	1	−5.04	3.57E-03

*FC* fold change, *FX* the population occurring in Fengxiang, *HL* the population occurring in the Helan Mountains, *NA* Not available

**Table 3:** Population genetics and expression data for the genes in the comparative set ‘HL vs HC’

Gene ID	Nr Description	Tajima’s D HL	Tajima’s D HC	Mean Fst	log_2_FC (HL vs HC)	*q* value
Cluster-41,210.51587	Venom serine protease 34-like	1.75	NA	0.45	3.76	6.58E-04
Cluster-41,210.75416	Cytochrome P450 6BQ2	0.96	1.25	−0.13	−2.92	1.33E-02
Cluster-41,210.35732	Cytochrome P450 6BQ4	0	0	0.57	−2.71	4.55E-02
Cluster-41,210.105304	ABCC4	0	0	0.16	−3.12	3.93E-03
Cluster-41,210.111638	Solute carrier family 25 member 36	0	0	−0.50	−6.11	5.49E-03
Cluster-41,210.145415	Solute carrier family 12 member 2, partial	NA	1.03	0.63	2.75	7.47E-03
Cluster-41,210.101334	Heat shock protein cognate 3	−1.13	NA	0.80	2.91	5.26E-03
Cluster-41,210.74253	Heat shock protein 67B2	NA	0	0.25	−3.25	1
Cluster-41,210.3937	Heat shock 70 kDa protein cognate 4	NA	0	8.33E-17	2.59	0.45

*FC* fold change, *HC* the population occurring in Hancheng, *HL* the population occurring in the Helan Mountains, *NA* Not available

### Enrichment pathway analysis of DEGs

We mapped all the DEGs to terms in the KEGG database. KEGG pathways with *P*-value < 0.05 between the two pairwise comparisons, i.e., ‘HL vs HC’ and ‘HL vs FX’, are provided in Additional file 1: Table S11. In the comparison of ‘HL vs HC’, ‘ABC transporters’ and ‘pancreatic secretion’ pathways were up-regulated, while ‘citrate cycle (TCA cycle)’, ‘fat digestion and absorption’, and ‘insulin resistance’ were down-regulated. In the comparison of ‘HL vs FX’, ‘ABC transporters’ were up-regulated, while ‘insulin signaling pathway’, ‘glucagon signaling pathway’ and ‘vitamin digestion and absorption’ were down-regulated.

In both pairwise comparisons of ‘HL vs HC’ and ‘HL vs FX’, the strongest changes in the top 20 GO categories were ‘tRNA (m1A) methyltransferase complex’, ‘methyltransferase complex’, ‘tRNA methyltransferase complex’ of cellular component (CC) and ‘tRNA methylation’ of biological process (BP) (Additional file 13: Figure S5). These four changes were significant enriched, with –log_2_(*P*-value) higher than 16 in ‘HL vs FX’. Among the top 20 of the strongest GO categories, eleven were common in ‘HL vs HC’ and ‘HL vs FX’ (Additional file 13: Figure S5).

### Validation of transcriptome data using qPCR

**Fig. 7:**
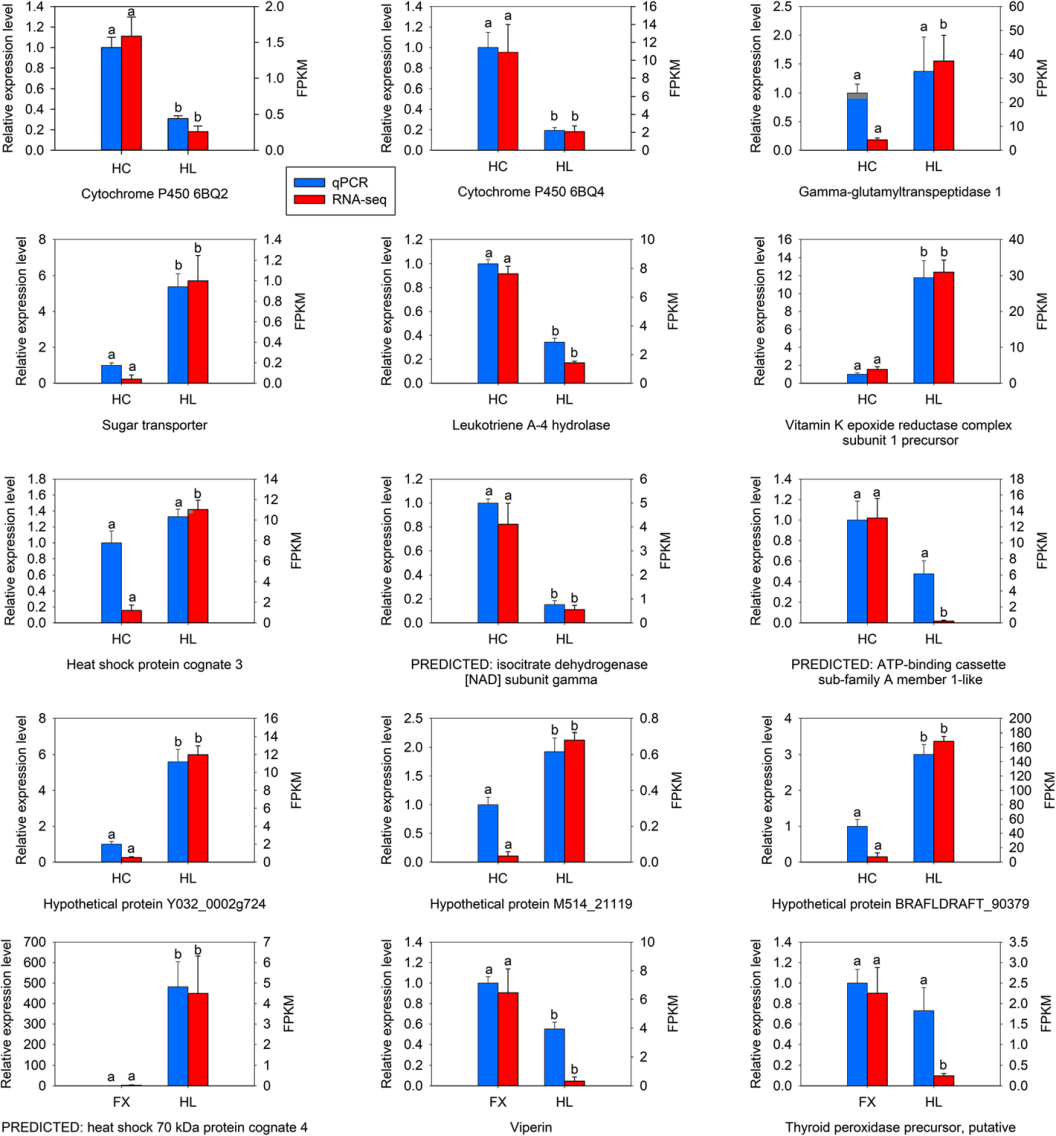
Gene expression levels of differentially expressed genes through RNA-seq and qRT-PCR analysis. FPKM: fragments per kilobase of transcript per million fragments mapped; FX: the population occurring in Fengxiang; HC: the population occurring in Hancheng; HL: the population occurring in the Helan Mountains

To validate the RNA-seq results, the relative expression levels of 15 selected genes were analysed by RT-qPCR, including 12 DEGs in ‘HL vs HC’ and three DEGs in ‘HL vs FX’. Among these 15 different expression genes, the majority exhibit a consistent expression pattern between RNA-Seq and qPCR (Fig. 7), indicating that RNA-seq data are reliable.

## Discussion

RNA-Seq is revolutionizing the fields of ecological and evolutionary genomics, and contributing new insights into the responses that organisms deploy in alternative environments (e.g., plant hosts) [12]. This approach has been employed to evaluate the genetic changes, from a broad view and at the level of candidate genes, in response to different host plants in insects [9, 11, 12, 33, 34]. In the present study, the strongest gene expression differences were revealed in the pairwise comparison between ‘HL vs HC’ (1826 DEGs), followed by the comparison between ‘HL vs FX’ (723 DEGs), while the weakest expression differences were found in the comparison between ‘HC vs FX’ (183 DEGs) (Fig. 3a). This is in line with the genetic differentiation among populations of *S. yangi* based on mitochondrial (*COI* + *COII* + *Cytb* + *A6A8*) genes, where the mean Fst among the three comparisons, ‘HL vs HC’, ‘HL vs FX’ and ‘HC vs FX’ was 0.0735, 0.0676 and 0.0121, respectively [8]. This suggests that differential gene expression in the midgut among these populations is closely associated with genetic variations of related populations.

The HC and FX populations of *S. yangi* specialize on *Z. jujuba* var. *spinosa*, while the HL population has shifted to chemically divergent *E. lepidosperma*. The two host plants differ in biochemical composition and, thus, are expected to have a different nutritional value for *S. yangi*. Our results show that the genes related to digestion were mainly up-regulated in the HL population when compared with the HC and FX populations (Fig. 4). Our results might indicate that the DEGs related to digestion were involved in adaptation to host shift in *S. yangi.*

Some differentially expressed metabolism-related genes of insects in adapting to different biochemical composition of host plants have been revealed [9, 12, 34]. For example, Chikate et al. [35] chose a set of trypsin and chymotrypsin transcripts and inspected their pattern of expression of the cotton bollworm *Helicoverpa armigera* in response to host plants with different nutritional qualities. It is suggested that serine proteases are key enzymes allowing this lepidopteran pest to adapt to many different host plants and, therefore, augmenting polyphagy [35]. Celorio-Mancera et al. [33] reached similar conclusions in the butterfly larvae of *Polygonia c-album.* In our present study, we also reveal that four genes (Cluster-41,210.32990, Cluster-41,210.95944, Cluster-41,210.113279, Cluster-41,210.51587) encoding serine proteases were differentially expressed in the HL population when compared with the HC and FX populations (Fig. 4; Additional file 10: Table S8), which may be involved in host selection and adaptation of *S. yangi*.

Besides the nutritional requirements, insects should at the same time be able to cope with toxic chemicals from their host plants. When confronted with host shift, insects can successfully survive on their new host plant through enzymes coded by detoxifying genes such as P450s, GSTs, UGTs and CEs [9, 11, 12, 33, 34]. From the midgut of *S. yangi*, besides P450s, we also found that five UGTs, six CEs and one GST were differentially expressed in the HL population when compared with the HC and FX populations*.* In addition to detoxifying enzymes, ABC transporters are also involved in detoxification [36], which are membrane-bound proteins involved in the movement of various substrates, including drugs and insecticides, across the lipid membrane [37]. The ABC superfamily is currently divided into eight distinct subfamilies (A–H) [38]. Among which, three subfamilies, ABCB, ABCC and ABCG, are involved in drug resistance [39]. In our study, differentially expressed ABC transporters were also detected, including ABCB8, ABCC1, ABCC4, ABCC5 and ABCG4, and majority of them were up-regulated in the HL population when compared with the HC and FX populations (Fig. 5; Additional file 11: Table S9). This suggests that these genes may play an important role in detoxification during the host plant shift of this cicada species. Besides the abovementioned proteins that are involved in detoxification, alkaline phosphatase is also associated with removal of alkaloids in pests, and high levels of alkaline phosphatase have been implicated in insecticide resistance [40, 41]. In our study, we found that an *alkaline phosphatase, tissue-nonspecific isozyme-like* gene (Cluster-41,210.64298) was exclusively expressed in the HL population (Fig. 5; Additional file 11: Table S9), indicating this gene may play a significant role in alkaloid resistance when *S. yangi* feeds on *E. lepidosperma*.

Reactive oxygen species (ROS) are derived from the metabolism of molecular oxygen [42], such as superoxide radicals (O_2_^−^), hydroxyl radical (OH^−^), H_2_O_2_, and hydroperoxides (ROOH), which are generated by exogenous and endogenous sources [43]. Exogenous sources, for example prooxidant allelochemicals produced by host plants, pose a serious challenge to herbivorous insects during insect–plant interactions. However, some herbivorous insects have evolved a midgut-based defense against the toxic effects of ROS [44, 45]. They can produce many enzymes for detoxifying ROS, including glutathione peroxidase (GPX), catalase (CAT), superoxide dismutase (SOD) and ascorbate peroxidase [46]. Mittapalli et al. [44] suggested that the induced expression of antioxidant genes, such as phospholipid glutathione peroxidases, catalases and superoxide dismutases in the midgut of a major insect pest of wheat worldwide, the Hessian fly *Mayetiola destructor*, to be a protective response to ROS ingested during feeding or food processing. Zhu et al. [47] also suggested that peroxidase potentially involved in insecticide metabolic resistance in the diamondback moth *Plutella xylostella*. In our present study, two DEGs encoding peroxidase were identified between the populations of *S. yangi* utilizing different host plants (Fig. 6; Additional file 12: Table S10), suggesting that they may also play an important role in protective response to ROS ingested during feeding or food processing.

When organisms are exposed to heat, cold or some other stresses, they can synthesize Hsps which participate in unfolding and relocalization of proteins damaged by the stresses [48]. Although a number of investigations have confirmed the importance of Hsps in resistance towards heat, cold and a range of other stresses, e.g., insecticides, heavy metals, desiccation, diseases, parasites and inbreeding, the most important role of Hsps is in response to stress resistance and adaptation to the environment [49]. However, Hsps expression confers some costs to fertility/fecundity, energy, development and survival [50, 51]. Therefore, organisms do not express Hsps unless environmental temperatures become severe, and their ability to induce Hsps is usually related to temperature conditions in their habitats [52]. For example, the limpet *Collisella scabra* inhabiting the high intertidal region has a greater tolerance to high temperatures and produces Hsps at a higher temperature than the related species *C. pelta* inhabiting the more sheltered upper midtidal region [53]. Some species of the freshwater coelenterate hydra *Hydra* from stable environments lack an ability to induce Hsps, while other related species from fluctuating environments exhibit heat shock response [54]. In addition, Bosch et al. [54] suggested that the ability of organisms to live in stressful habitats might be related to the ability to produce Hsps in response to stress, and that different organisms may utilize the same Hsp in different manners or produce different Hsps in response to thermal stress in different habitats. In our present study, we found that five unigenes encoding Hsps were differentially expressed in the HL population when compared with the HC and FX populations (Fig. 6; Additional file 12: Table S10). The habitats of the HL population, surrounded by deserts or semi-deserts, occupy at higher latitude and higher altitude than that of the HC and FX populations (Table 4). Hsps may play an important role in response to temperature stress and adaptation to the desert or semi-desert environments in the HL population of *S. yangi*.

**Table 4:** Host plants and locality data of *S. yangi* investigated in this study

Population	Location	Host plant	Latitude	Longitude	Elevation (m)
HL	The Helan (HL) Mountains, Ningxia Hui Nationality Autonomous Region	*Ephedra lepidosperma* C. Y. Cheng	38°33.699′N	105°55.217′E	1400–1600
HC	Hancheng (HC), Shaanxi	*Ziziphus jujuba* Mill. var. *spinosa* (Bunge) Hu ex H. F. Chow	35°31.683′N	110°25.927′E	582
FX	Fengxiang (FX), Shaanxi	*Z. jujuba* var. *spinosa*	34°31.273′N	107°24.044′E	800

Animals living in desert environments are forced to endure intense heat and drought, and as a result, species living in deserts likely involves a large number of adaptive traits, e.g., the ability to maintain the proper water and solute balance [55, 56]. Aquaporins (AQP), a large protein family facilitate transport of water between cells [57] and maintain water balance in the kidney [58–60]. For example, AQP2 transports water back into the kidney from the collecting duct [61, 62] and plays an essential role in the urinary concentrating mechanism in mammals. Mice lacking AQP2 suffer severe polyuria, with average basal daily urine volumes approximately equivalent to body weight and are totally unable to concentrate urine [63]. Similarly, mice lacking AQP1 and AQP4 also presented defects in water reabsorption [63]. While AQP4 is apparently absent in desert species such as kangaroo rats [64] and camelids [65]. In our study, an *aquaporin AQPAe.a* gene (Cluster-41,210.118935) and an *aquaporin 2-like* gene (Cluster-41,210.63368) were up-regulated in the HL population when compared with the HC and FX populations (Fig. 6; Additional file 12: Table S10). This suggests that they may play an important role in response to water deprivation for the HL population which occurs in the desert or semi*-*desert environments of the Helan Mountains. Regarding solute balance, another gene family, solute carriers (Slc), are responsible for the maintenance of electrolyte levels [66, 67]. Several studies have revealed that some members of solute carriers play a significant role in the osmoregulation in desert animals [56, 68–70]. For example, solute carrier family 2 member 9 (Slc2a9) has been suggested to be undergoing positive selection in kidney transcriptomes in the desert-adapted mouse *Peromyscus eremicus* [69]. A study of differential gene expression in the cactus mouse *P. eremicus* revealed that Slc2a1 and Slc8a1 showed responses to acute dehydration [56]. Recently, Giorello et al. [71] considered Slc12a2 may be related to water conservation in desert animals. In our present study, a unigene encoding Slc12a2 was up-regulated in the HL population (Fig. 6; Additional file 12: Table S10), suggesting that it may also be related to water conservation in the desert or semi*-*desert environments.

Host shift and subsequent adaptation can drive evolution and diversification of plant-feeding insects [9, 72]. In the context of a host switch, transcriptional plasticity in metabolic pathways involved in processing and detoxifying food resources may produce a phenotype that is well matched to the new environment (e.g., the deserts or semi*-*deserts). Directional selection is expected to further alter the plastic response in the direction of the optimum, resulting in adaptive evolution (known as the Baldwin effect) [73]. In this case, the DEGs detected in the midgut of *S. yangi* might be initially beneficial in the process of host shift in this species, which may subsequently evolve in a long-term feeding on a new host plant (viz., *E. lepidosperma*). A previous study indicated that the HL population has evolved into a distinct independent lineage according to the analysis of population differentiation based on both molecular data and males’ calling song structure [8]. The deserts or semi-deserts surrounding the Helan Mountains were previously shown as a major dispersal and climatic barrier for gene flow of related animals [74]. Such barriers appear to have promoted divergence of the HL lineage from other lineages of *S. yangi* [8]. Separated populations are potentially to become distinct species, as separated populations are seldom to get reunited after long-term chance and selection reciprocity, which may cause such populations to diverge and enhance many traits associated with reproductive isolation [75]. Differences in the patterns of gene expression in pairwise comparisons of ‘HL vs HC’ and ‘HL vs FX’ may be representative of the genetic changes which facilitate the colonization of novel habitats for *S. yangi*, and help to mitigate the stresses imposed by a novel host containing different xenobiotics.

Differential gene expression could be affected by several factors, such as genetic variations between populations [71], response to different host plants [9, 11, 12], response to abiotic factors (i.e., temperature) [76–78], or response to different microbiota in the alimentary canal [79–82]. Our analysis revealed that a certain number of genes were differentially expressed among populations utilizing different host plants and living in different habitats. Furthermore, the weakest expression differences were found between the populations utilizing the same host plant and occurring in the closest habitats. Given that the HL population is locally specialized on the endemic host plant *E. lepidosperma* but other populations specialize on *Z. jujuba* var. *spinosa*, results of our present study suggest that host shift could, coupled with geographical barriers (i.e., the deserts or semi*-*deserts surrounding the Helan Mountains) and ecological changes, have acted as effective factors resulting in the population divergence and allopatric speciation in *S. yangi*. Further study is required to confirm that differential expression of the various DEGs identified in our comparative analysis of cicada transcriptomes is due to the mechanisms of adaptation to the novel host plant and new habitats.

## Conclusions

In this study, we investigated the transcriptional changes in the midgut of the cicada *Subpsaltria yangi*. Our results indicate that the DEGs related to digestion, detoxification, oxidation-reduction, stress response, water-deprivation response and osmoregulation may be important in the adaptation to host plants and habitats by *S. yangi*. The observed differences in gene expression among *S. yangi* populations occurring in different habitats and utilizing different host plants may help explain the metabolic mechanism of adaptation in *S. yangi* populations to a novel host plant and drier habitats, and inform future studies on the molecular mechanisms of population divergence or speciation in herbivorous insects.

## Methods

### Field investigation of host plant utilization by *Subpsaltria yangi*

The host plant utilization by *S. yangi* was investigated during its summer emergence period. Specifically, during the behavioral ecology study of the population occurring in the Helan Mountains of Ningxia Hui Nationality Autonomous Region from May to June, 2016 and 2017, virgin adults of *S. yangi* were captured at night or in the early morning when they emerged from the soil. We simultaneously recorded what host plant the insects fed on in habitats of the Helan Mountains where *Z. jujuba* var. *spinosa* and *E. lepidosperma* are both available. For the populations from Hancheng and Fengxiang, Shaanxi Province, the host plant utilization of adults was investigated during specimen collection at the same season in 2016 and 2017.

### Insect collection and dissection

Transferring cicada nymphs to new roots is a difficult process and results in high mortality [83, 84]. Adult cicadas are also difficult to rear in the laboratory. Thus, adults collected from wild populations were used in this study. The plant *E. lepidosperma* is an endemic species to the Helan Mountains [19]. It is very common that many habitats in the Helan Mountains only contain the host plant *E. lepidosperma* for *S. yangi*, although the host plant *Z. jujuba* var. *spinosa* mixes with the former in a few habitats*.* To obtain representative *S. yangi* samples exclusively feeding on *E. lepidosperma*, *S. yangi* adults from the Helan Mountains were collected at the habitats that only contain *E. lepidosperma* in June, 2016. In order to obtain representative *S. yangi* samples exclusively feeding on *Z. jujuba* var. *spinosa*, adults from Hancheng feeding on *Z. jujuba* var. *spinosa* were collected in June, 2016. Due to the less controlled conditions of the two populations, another population feeding on *Z. jujuba* var. *spinosa* in Fengxiang that has a similar habitat to the HC population was also used to strengthen the results, which was collected in June, 2016 (Table 4).

Collected adults from the three localities were reared on their corresponding host plants and transferred alive to the laboratory for dissection and RNA extraction. Midguts of the adults were dissected on ice in sterile phosphate buffered saline (PBS) treated with 0.1% diethylpyrocarbonate (DEPC) under an Olympus SZX 10 stereomicroscope (Olympus Corporation, Tokyo, Japan), and washed in sterile ice-cold PBS to remove the sap of host plants, then were immediately frozen and stored at − 80 °C for subsequent RNA extraction.

### RNA extraction, cDNA library preparation and Illumina sequencing

For transcriptome sequencing, one sample included the midgut from one adult of *S. yangi* was collected. Total RNA was extracted with Trizol reagent (Life Technologies, USA) according to the manufacturer’s instructions. DNA contaminants were removed by treating RNA extraction products with RNase-free DNase (Ambion, Austin, TX, USA), and then were purified through phenol-chloroform extraction. RNA quality was examined using 1% agarose gel and the concentration was determined using NanoDrop (Thermo). In total, nine RNA samples (3 populations × 3 replicated samples) were obtained. Sequencing libraries were constructed using NEBNext® Ultra™ RNA Library Prep Kit for Illumina® (NEB, USA) following manufacturer’s recommendations, and index codes were added to attribute sequences to each sample. In brief, mRNA was purified from total RNA using poly-T oligo-attached magnetic beads and then was fragmented. Double-stranded cDNAs were then synthesized using reverse transcriptase (Superscript II, Life Technologies) and random hexamer primers. The standard Illumina protocol was followed thereafter to develop mRNA-seq libraries. Subsequently, the library preparations were sequenced on an Illumina Hiseq platform, and 150 bp paired-end reads were generated. All sequencing data were deposited in the NCBI and can be accessed in the Short Read Archive (SRA) under accession number SRP100344.

### Data processing assembly and annotation

Raw reads in FASTQ format were processed through in-house Perl scripts. Clean reads were obtained by removing reads containing adapter, reads containing poly-N and low quality reads from the raw reads. At the same time, Q20, Q30, GC-content and sequence duplication level of the clean data were calculated. The sequenced left files (read1 files) from the nine samples were pooled into one big left.fq file, and right files (read2 files) into one big right.fq file. Transcriptome assembly was accomplished based on the left.fq and right.fq using Trinity (version r20140413p1) [85] with min_kmer_cov set to 2 and all other parameters set as default. The completeness of the assembly was assessed with the Benchmarking Universal Single-Copy Orthologs (BUSCO, version 3.0.2) tool [86] using the insecta_odb9 dataset. The longest transcript of each gene was defined as the ‘unigene’ for functional annotation. Seven databases were used to annotate the gene function: Nr (NCBI non-redundant protein sequences, NCBI blast 2.2.28+, E-value = 1E-5); Nt (NCBI nucleotide sequences, NCBI blast 2.2.28+, E-value = 1E-5); Pfam (Protein family, http://pfam.xfam.org/, HMMER 3.0 package, hmmscan, E-value = 0.01); KOG/COG (Clusters of Orthologous Groups of proteins, http://www.ncbi.nlm.nih.gov/COG/, NCBI blast 2.2.28+, E-value = 1E-3); Swiss Prot (a manually annotated and reviewed protein sequence database, http://www.ebi.ac.uk/uniprot/, NCBI blast 2.2.28+, E-value = 1E-5); KO (KEGG Ortholog database, http://www.genome.jp/kegg/, KAAS (version r140224), KEGG Automatic Annotation Server, E-value = 1E-10) [87]; and GO (Gene Ontology, http://www.geneontology.org/, Blast2GO v2.5, E-value = 1E-6) [88].

### Analysis of gene expression patterns

The read counts of a gene in each sample were estimated by mapping clean reads to the Trinity transcripts assembled by RSEM (version v1.2.15, bowtie2 default parameters, mismatch = 0) [89]. FPKM (fragments per kilobase of transcript per million fragments mapped) was used to estimate the level of gene expression [90]. Differential gene expression analysis among conditions/groups was performed using the DEGseq R package (version 1.10.1) with the threshold q-value < 0.05 and absolute Fold Change > 2 demarcating significantly different expression levels [91]. We evaluated genetic differentiation through the Fst fixation index and different selection regimens using Tajima’s D statistic. Fst and Tajima’s D statistics for sliding windows were calculated using VCFtools [92], with a window of 40,000. Venn diagram analysis of DEGs between different groups was performed using the OmicShare tools, a free online platform for data analysis (http://www.omicshare.com/tools/index.php/Home/Soft/venn). Hierarchical clustering and Venn diagram were used to illustrate the differential gene expression patterns between different groups, which were carried out using the OmicShare tools, a free online platform for data analysis (www.omicshare.com/tools). Heatmaps were also produced using the OmicShare tools (Z-score normalization set to row, and all other parameters set as default) based on normalized FPKM data (http://www.omicshare.com/tools/Home/Soft/heatmap).

### Enrichment analysis of GO enrichment and KEGG pathway

GO term enrichment analysis of DEGs was carried out using the GOseq R package (version 1.10.0) which can adjust for gene length bias in DEGs based on the Wallenius non-central hyper-geometric distribution [93]. KEGG is a database resource for understanding high-level functions and relationships among biological systems, such as the cell, organism, and ecosystem, from molecular-level information, which are represented by large-scale molecular datasets generated by genome sequencing and other high-throughput experimental technologies (http://www.genome.jp/kegg/). Here, KOBAS (KEGG Orthology-Based Annotation System) (version v2.0.12) [94] was used to test the statistical enrichment of DEGs in KEGG pathways.

### Real-time quantitative PCR analysis

In order to validate the results from our transcriptome sequencing analysis, the relative expression levels of 15 selected genes were confirmed by reverse transcription quantitative PCR (RT-qPCR). RNA was isolated from midguts and reverse-transcribed to cDNA using PrimeScript II 1st strand cDNA synthesis kit (Takara, Dalian, China). The gene-specific primers were designed using the predicted CDSs as reference sequences. All the primers are listed in Additional file 1: Table S12. RT-qPCR reactions were prepared with the SYBR Premix Ex Taq™ Kit (Takara), following the manufacturer’s instruction. Quantitative reactions were performed on the Real-Time PCR Detection System (ABI 7500, Applied Biosystems, USA). The qPCR cycling parameters were as follows: 95 °C for 5 min, followed by 40 cycles of 95 °C for 10 s and 60 °C for 30 s, melt curves stages at 95 °C for 15 s, 60 °C for 1 min, and 95 °C for 15 s. Negative controls without template were included in each experiment. To check reproducibility, the qPCR reaction for each sample was performed in three technical replicates and three biological replicates. The relative gene expression was calculated using the 2^–△△Ct^ method [95]. Two reference genes, Ribosomal protein L9 and Actin, were used for normalization. Data analysis was performed using the SPSS Statistics 20.0 software (IBM SPSS Statistics Inc., Chicago, IL, USA). Results are reported as mean ± SE.

## Additional files

Additional file 1: Table S1–S3, Table S11 and Table S12. **Table S1.** Number of paired reads obtained by RNA-Seq. **Table S2.** Annotation of unigenes in different databases. **Table S3.** Species distribution is shown as the percentage of the total homologous sequences. **Table S11.** List of KEGG pathways with *P*-value < 0.05 between the two pairwise comparisons, ‘HL vs HC’ and ‘HL vs FX’. **Table S12.** Primers used in qRT-PCR (DOC 137 kb)

Additional file 2: **Figure S1.** Gene ontology classification of assembled unigenes. The 59,179 matched unigenes were classified into three functional categories: molecular function, biological process and cellular component (TIF 775 kb)

Additional file 3: **Figure S2.** KOG functional classification of all unigenes. A total of 28,896 unigenes showed significant similarity to the sequences in KOG databases and were clustered into 26 categories (TIF 754 kb)

Additional file 4: **Figure S3.** KEGG pathway distributions of midgut unigenes. The genes according to KEGG metabolic pathway involved was divided into five branches: A, Cellular processes; B, Environmental information processing; C, Genetic information processing; D, Metabolism; E, Organismal systems (TIF 1056 kb)

Additional file 5: **Table S4.** Primers designed for detecting simple sequence repeats (SSRs) in genes of *Subpsaltria yangi (XLS 7778 kb)*

Additional file 6: **Figure S4.** Cluster analysis of differentially expressed genes. Different colors indicate different levels of gene expression: from red to blue, the log_10_(FPKM + 1) value ranges from large to small. FX: the population occurring in Fengxiang; HC: the population occurring in Hancheng; HL: the population occurring in the Helan Mountains (TIF 695 kb)

Additional file 7: **Table S5.** Annotation of differentially expressed genes in the comparison of ‘HC vs FX’. FX: the population occurring in Fengxiang; HC: the population occurring in Hancheng (XLS 286 kb)

Additional file 8: **Table S6.** Annotation of differentially expressed genes in the comparison of ‘HL vs HC’. HC: the population occurring in Hancheng; HL: the population occurring in the Helan Mountains (XLS 2152 kb)

Additional file 9: **Table S7.** Annotation of differentially expressed genes in the comparison of ‘HL vs FX’. FX: the population occurring in Fengxiang; HL: the population occurring in the Helan Mountains (XLS 895 kb)

Additional file 10: **Table S8.** Candidate DEGs related to digestion (XLS 45 kb)

Additional file 11: **Table S9.** Candidate DEGs related to detoxification and oxidation-reduction (XLS 71 kb)

Additional file 12: **Table S10.** Candidate DEGs related to stress response, water-deprivation response and putative osmoregulation (XLS 82 kb)

Additional file 13: **Figure S5.** GO enrichment of differentially expressed genes in (**a**) ‘HL vs HC’ and (**b**) ‘HL vs FX’. The 20 most enriched GO terms are shown together with their –log_2_(*P*-value) and number of genes (adjacent the bars). FX: the population occurring in Fengxiang; HC: the population occurring in Hancheng; HL: the population occurring in the Helan Mountains (TIF 1528 kb)

## Abbreviations

A6A8: ATPase subunits
ABC transporters: ATP-binding cassette transporters
AQP: Aquaporin
BP: Biological process
BUSCO: Benchmarking universal single-copy orthologs
CAT: Catalase
CC: Cellular component
cDNA: Complementary DNA
CDS: Coding sequence
CE: Carboxylesterases
COI: Cytochrome c oxidase subunit I
COII: Cytochrome c oxidase subunit II
Cytb: Cytochrome b
DEGs: Differentially expressed genes
DEPC: Diethylpyrocarbonate
DNA: Deoxyribonucleic acid
FC: Fold change
FPKM: Fragments per kilobase of transcript per million fragments mapped
Fst: Fixation index
FX: Fengxiang
GO: Gene Ontology
GPX: Glutathione peroxidase
GST: Glutathione S-transferase
HC: Hancheng
HL: Helan Mountains
Hsp: Heat shock proteins
KAAS: KEGG automatic annotation server
KEGG: Kyoto encyclopedia of genes and genomes
KO: KEGG ortholog database
KOBAS: KEGG orthology-based annotation system
KOG: Eukaryotic orthologous groups
NCBI: National center for biotechnology information
Nr: NCBI non-redundant protein sequences
Nt: NCBI nucleotide sequences
P450: Cytochrome P450 monooxygenases
PBS: Phosphate buffered saline
PCR: Polymerase chain reaction
Pfam: Protein family
RNA: Ribonucleic acid
RNA-Seq: RNA sequencing
ROS: Reactive oxygen species
RT-qPCR: Real-time quantitative PCR
Slc: Solute carriers
SOD: Superoxide dismutase
SPSS: Statistical package for the social sciences
SRA: Short read archive
SSR: Simple sequence repeats
TCA cycle: Tricarboxylic acid cycle
tRNA: Transfer RNA
UGT: UDP-glycosyltransferases
