# Supplementary material for: De novo comparative transcriptome analysis of a rare cicada, with identification of candidate genes related to adaptation to a novel host plant and drier habitats
Source: BMC Genomics. 2019 Mar 7;20:182. doi: 10.1186/s12864-019-5547-y (PMC6407286; doi:10.1186/s12864-019-5547-y)
Supplement: Supplementary file 1 — Table S1–S3, Table S11 and Table S12. Table S1. Number of paired reads obtained by RNA-Seq. Table S2. Annotation of unigenes in different databases. Table S3. Species distribution is shown as the percentage of the total homologous sequences. Table S11. List of KEGG pathways with P-value < 0.05 between the two pairwise comparisons, ‘HL vs HC’ and ‘HL vs FX’. Table S12. Primers used in qRT-PCR (DOC 137 kb) [file 12864_2019_5547_MOESM1_ESM.doc]

**Table S1. Number of paired reads obtained by RNA-Seq**

| Sample | Raw Reads | Clean Reads | Clean Bases | Error (%) | Q20 (%) | Q30 (%) | GC Content (%) |
| --- | --- | --- | --- | --- | --- | --- | --- |
| FX_1 | 48188814 | 46973576 | 7.05G | 0.01 | 97.68 | 94.03 | 36.24 |
| FX_2 | 60999150 | 59408788 | 8.91G | 0.01 | 97.44 | 93.43 | 35.72 |
| FX_3 | 48222116 | 46916582 | 7.04G | 0.02 | 97.36 | 93.28 | 35.24 |
| HL_1 | 53506032 | 51973160 | 7.8G | 0.02 | 96.61 | 91.55 | 36.77 |
| HL_2 | 72608676 | 70622700 | 10.59G | 0.01 | 97.41 | 93.33 | 36.84 |
| HL_3 | 43618514 | 42277572 | 6.34G | 0.01 | 97.43 | 93.37 | 37.21 |
| HC_1 | 46518408 | 45334890 | 6.8G | 0.01 | 97.44 | 93.4 | 36.41 |
| HC_2 | 50906400 | 49630480 | 7.44G | 0.01 | 97.44 | 93.42 | 35.72 |
| HC_3 | 56718818 | 55249466 | 8.29G | 0.02 | 97.33 | 93.17 | 36.49 |

**Table S2. Annotation of unigenes in different databases**

| Database | No. of annotated unigenes | Percentage of annotated unigenes (%) |
| --- | --- | --- |
| Nr | 72548 | 30.9 |
| GO | 59179 | 25.2 |
| Pfam | 58793 | 25.1 |
| Swiss-Prot | 39526 | 16.8 |
| KOG | 28896 | 12.3 |
| KEGG | 22621 | 9.6 |
| Nt | 9855 | 4.2 |
| At least one | 89441 | 38.1 |
| All | 5392 | 2.3 |

**Table S3. Species distribution is shown as the percentage of the total homologous sequences**

| Species | Percentage of homologous hits |
| --- | --- |
| *Zootermopsis nevadensis* | 22.7% |
| *Acyrthosiphon pisum* | 9.2% |
| *Lasius niger* | 5.7% |
| *Tribolium castaneum* | 4.6% |
| *Diaphorina citri* | 4.5% |
| *Stegodyphus mimosarum* | 2.8% |
| *Athalia rosae* | 2.2% |
| *Bombyx mori* | 2.2% |
| *Pediculus humanus* | 2.1% |
| *Vollenhovia emeryi* | 2.1% |
| *Hydra vulgaris* | 2.0% |
| *Plutella xylostella* | 1.7% |
| *Megachile rotundata* | 1.5% |
| *Solenopsis invicta* | 1.4% |
| *Orussus abietinus* | 1.3% |
| *Cerapachys biroi* | 1.2% |
| *Riptortus pedestris* | 1.1% |
| *Linepithema humile* | 1.1% |
| *Camponotus floridanus* | 1.1% |
| *Harpegnathos saltator* | 1.0% |
| *Microplitis demolitor* | 1.0% |
| Others | 27.4% |

**Table S11. List of KEGG pathways with P-Value  <  0.05 between the two pairwise comparisons, HL vs HC and HL vs FX**

| Pathway term | Pathway ID | Input number | Background number | P-Value |
| --- | --- | --- | --- | --- |
| **HL vs HC** |  |  |  |  |
| **Pathways for up-regulated DEGs** |  |  |  |  |
| Pathogenic Escherichia coli infection | ko05130 | 8 | 130 | 0.004579249 |
| Shigellosis | ko05131 | 8 | 147 | 0.009031991 |
| Gap junction | ko04540 | 9 | 187 | 0.011971527 |
| Ras signaling pathway | ko04014 | 12 | 292 | 0.012773138 |
| Leukocyte transendothelial migration | ko04670 | 8 | 157 | 0.012835415 |
| Bacterial invasion of epithelial cells | ko05100 | 8 | 159 | 0.013718497 |
| Phospholipase D signaling pathway | ko04072 | 11 | 262 | 0.014533313 |
| Fatty acid biosynthesis | ko00061 | 4 | 49 | 0.017257877 |
| ABC transporters | ko02010 | 10 | 237 | 0.01868485 |
| Proteoglycans in cancer | ko05205 | 14 | 403 | 0.026755301 |
| Cytokine-cytokine receptor interaction | ko04060 | 3 | 33 | 0.029467333 |
| Regulation of actin cytoskeleton | ko04810 | 12 | 332 | 0.029896041 |
| Focal adhesion | ko04510 | 12 | 336 | 0.032237486 |
| Rap1 signaling pathway | ko04015 | 12 | 339 | 0.034077725 |
| Pancreatic secretion | ko04972 | 9 | 234 | 0.040398483 |
| Mismatch repair | ko03430 | 4 | 66 | 0.042284024 |
| Type I diabetes mellitus | ko04940 | 2 | 17 | 0.048427631 |
| MicroRNAs in cancer | ko05206 | 11 | 320 | 0.048723326 |
|  |  |  |  |  |
| **HL vs HC** |  |  |  |  |
| **Pathways for down-regulated DEGs** |  |  |  |  |
| Citrate cycle (TCA cycle) | ko00020 | 6 | 175 | 0.011063861 |
| Fat digestion and absorption | ko04975 | 4 | 85 | 0.013259595 |
| Insulin resistance | ko04931 | 8 | 298 | 0.014010212 |
|  |  |  |  |  |
| **HL vs FX** |  |  |  |  |
| **Pathways for up-regulated DEGs** |  |  |  |  |
| MAPK signaling pathway | ko04010 | 7 | 370 | 0.028348424 |
| ABC transporters | ko02010 | 5 | 237 | 0.040767698 |
|  |  |  |  |  |
| **HL vs FX** |  |  |  |  |
| **Pathways for down-regulated DEGs** |  |  |  |  |
| Calcium signaling pathway | ko04020 | 7 | 320 | 0.0010356 |
| Insulin signaling pathway | ko04910 | 7 | 349 | 0.00168206 |
| Glucagon signaling pathway | ko04922 | 6 | 324 | 0.005201609 |
| Vitamin digestion and absorption | ko04977 | 3 | 98 | 0.012584525 |
| Insect hormone biosynthesis | ko00981 | 2 | 40 | 0.017126554 |
| cGMP-PKG signaling pathway | ko04022 | 5 | 322 | 0.020597151 |
| Vascular smooth muscle contraction | ko04270 | 4 | 231 | 0.0264068 |
| Steroid hormone biosynthesis | ko00140 | 3 | 163 | 0.045264762 |
| Chemical carcinogenesis | ko05204 | 4 | 277 | 0.04594726 |

FX: the population occurring in Fengxiang; HC: the population occurring in Hancheng; HL: the population occurring in the Helan Mountains

**Table S12.** Primers used in qRT-PCR

| Gene ID | Gene name | Forward primer | Reverse primer |
| --- | --- | --- | --- |
| Cluster-41210.75416 | Cytochrome P450 6BQ2 | CATGTGCATTCCGTTCACCG | ACCGGCTATGCGGTTTGTTA |
| Cluster-41210.35732 | Cytochrome P450 6BQ4 | GATGACAATTCGTGCGACGG | CGGGTTTCTAGCGAGTTCGT |
| Cluster-41210.100678 | Gamma-glutamyltranspeptidase 1 | CGACTTTCACTTGCAGTGCC | GATGCGTCACGATTCAGTGC |
| Cluster-41210.46555 | Sugar transporter | TGACGGTTTCTCGCATTACT | AGTAGCCAGTTGCCTCCATT |
| Cluster-41210.84038 | Leukotriene A-4 hydrolase | GTAATTCGCGGCGCTTCAAT | CACAGGAACACCGTGGCTAT |
| Cluster-41210.103318 | Vitamin K epoxide reductase complex subunit 1 precursor | CGCCCTGGTTGTTGAAACAG | ACTGCAGCAGTCCAAAACCT |
| Cluster-41210.101334 | Heat shock protein cognate 3 | TACGTCGGAAAGCAACGGAG | TTTGGTGGGCGGTATGTTGT |
| Cluster-31368.0 | Isocitrate dehydrogenase [NAD] subunit gamma, mitochondrial-like isoform X2 | TGCATTCCGCTGGTACCAAT | GCACACGATAACTCCCGTCA |
| Cluster-41210.96483 | ATP-binding cassette sub-family A member 1-like | ACAACAGTGGCTTAATAGCTGGA | ATCTGTTTGTTGGAGAGTGTGTGTGT |
| Cluster-41210.144646 | Hypothetical protein Y032_0002g724 | ATCTTGAAGAGGGTGGGGGA | CAACAGGGGTACTCAGAGCA |
| Cluster-41210.38266 | Hypothetical protein M514_21119, partial | TGGCAGCAGTGGAAAACAGA | CAGATTCAGGAGCTCCCGTG |
| Cluster-41210.119000 | Hypothetical protein BRAFLDRAFT_90379 | TGTGTGTGTGTGTGTGTAAAGT | GGGAGTACAGTAACAAGATCCCA |
| Cluster-41210.3937 | Heat shock 70 kDa protein cognate 4 | GATGTCGCAACGATCATCGC | GTCGTTTTCAACCCAGCACC |
| Cluster-41210.86189 | Viperin | CGTGTTTGTGCCTGTCCAAG | AGATTGGTCGAGGACAGGGA |
| Cluster-41210.98787 | Thyroid peroxidase precursor, putative | GGCTATGTCAGTTGCCTGCA | CCCCCAAAATGGTAAAGCATTGT |
| Cluster-41210.106035 | 60S ribosomal protein L9 | CCCGTCTGCTGAAAGTGGAA | ACGCCTGGAGCCATCTTTAC |
| Cluster-41210.95612 | Actin | GTCTGCCTACGATGGATGGG | CCTTGGTGGTGGTGTTCACT |
