# Supplementary figures and images for: De novo comparative transcriptome analysis of a rare cicada, with identification of candidate genes related to adaptation to a novel host plant and drier habitats
Source: BMC Genomics. 2019 Mar 7;20:182. doi: 10.1186/s12864-019-5547-y (PMC6407286; doi:10.1186/s12864-019-5547-y)

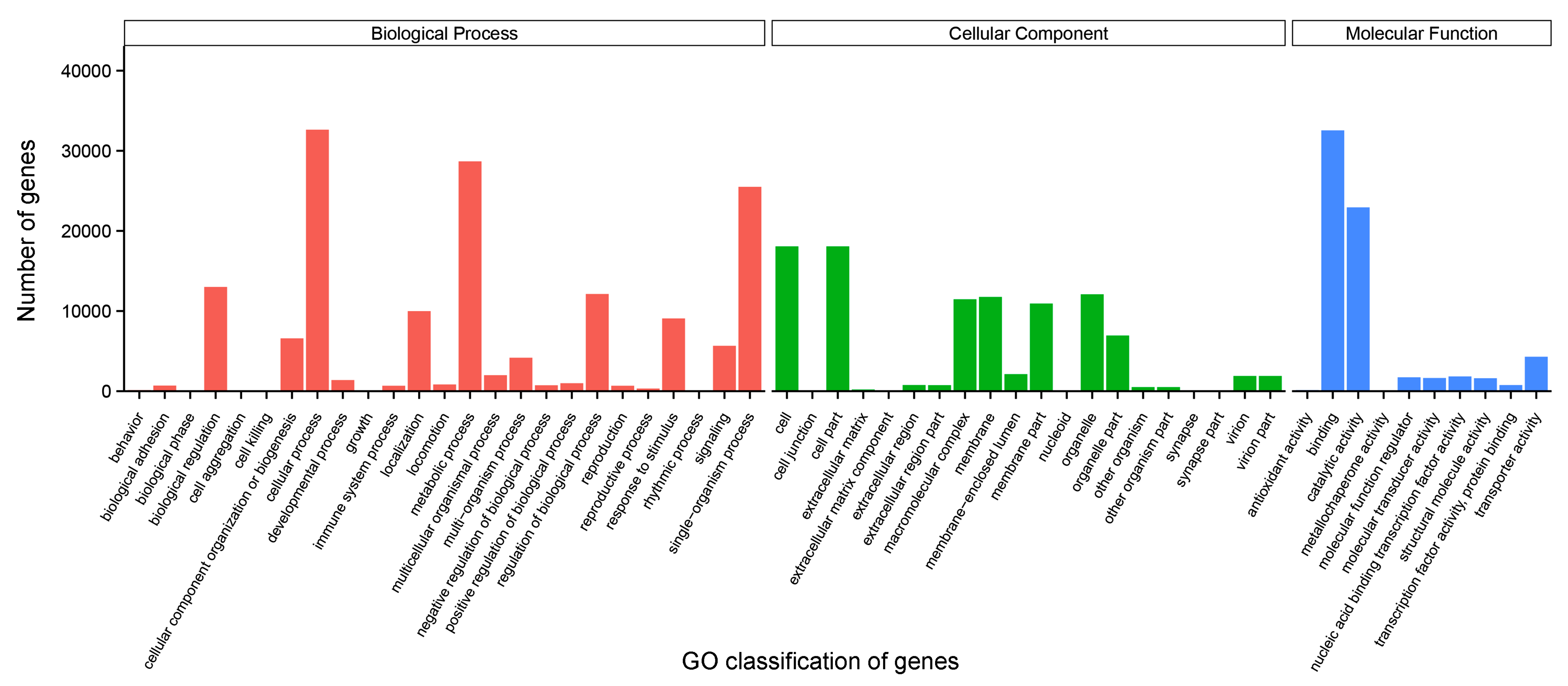

Supplement: Supplementary file 2 — Figure S1. Gene ontology classification of assembled unigenes. The 59,179 matched unigenes were classified into three functional categories: molecular function, biological process and cellular component (TIF 775 kb) [file 12864_2019_5547_MOESM2_ESM.tif]

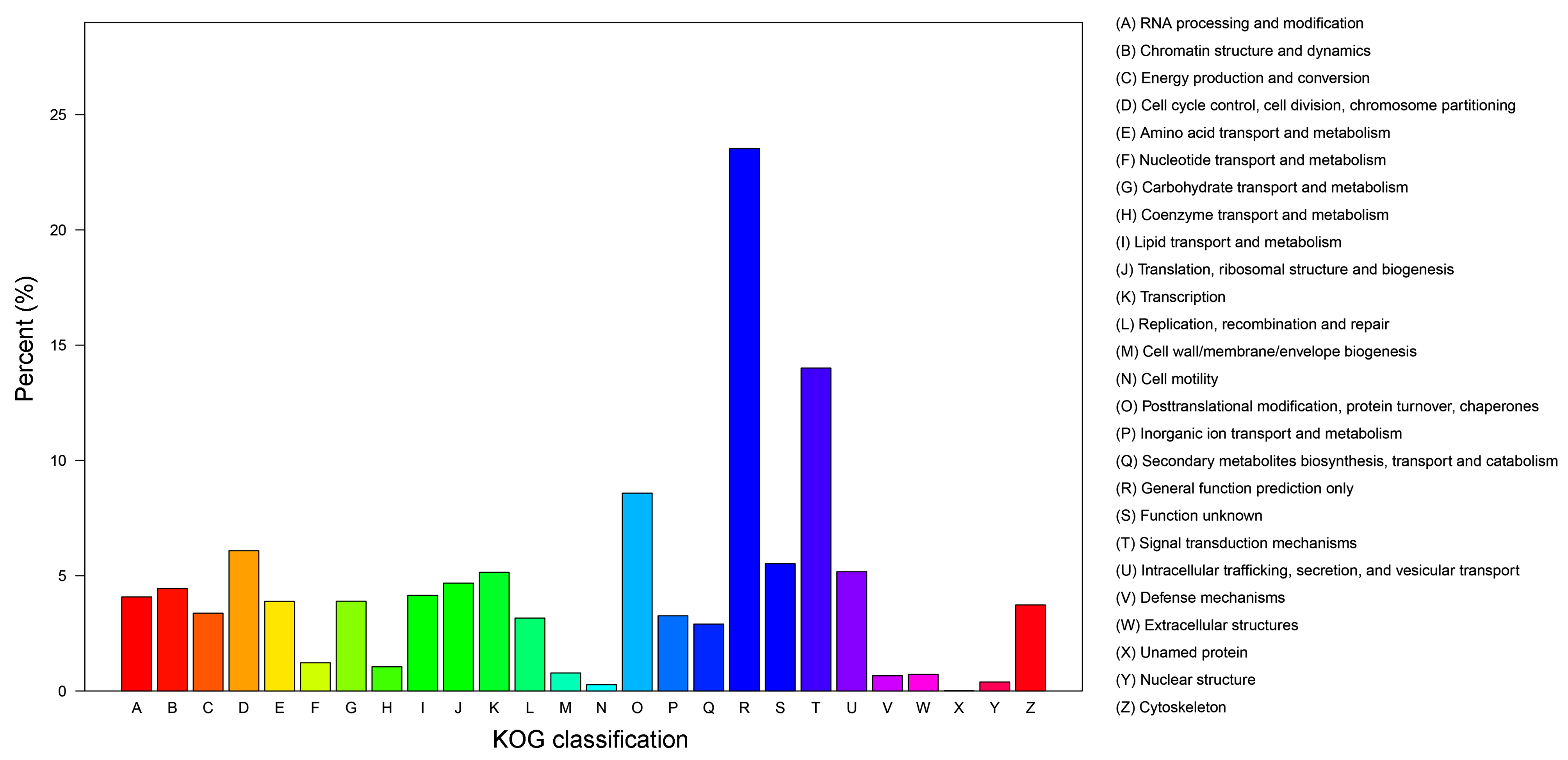

Supplement: Supplementary file 3 — Figure S2. KOG functional classification of all unigenes. A total of 28,896 unigenes showed significant similarity to the sequences in KOG databases and were clustered into 26 categories (TIF 754 kb) [file 12864_2019_5547_MOESM3_ESM.tif]

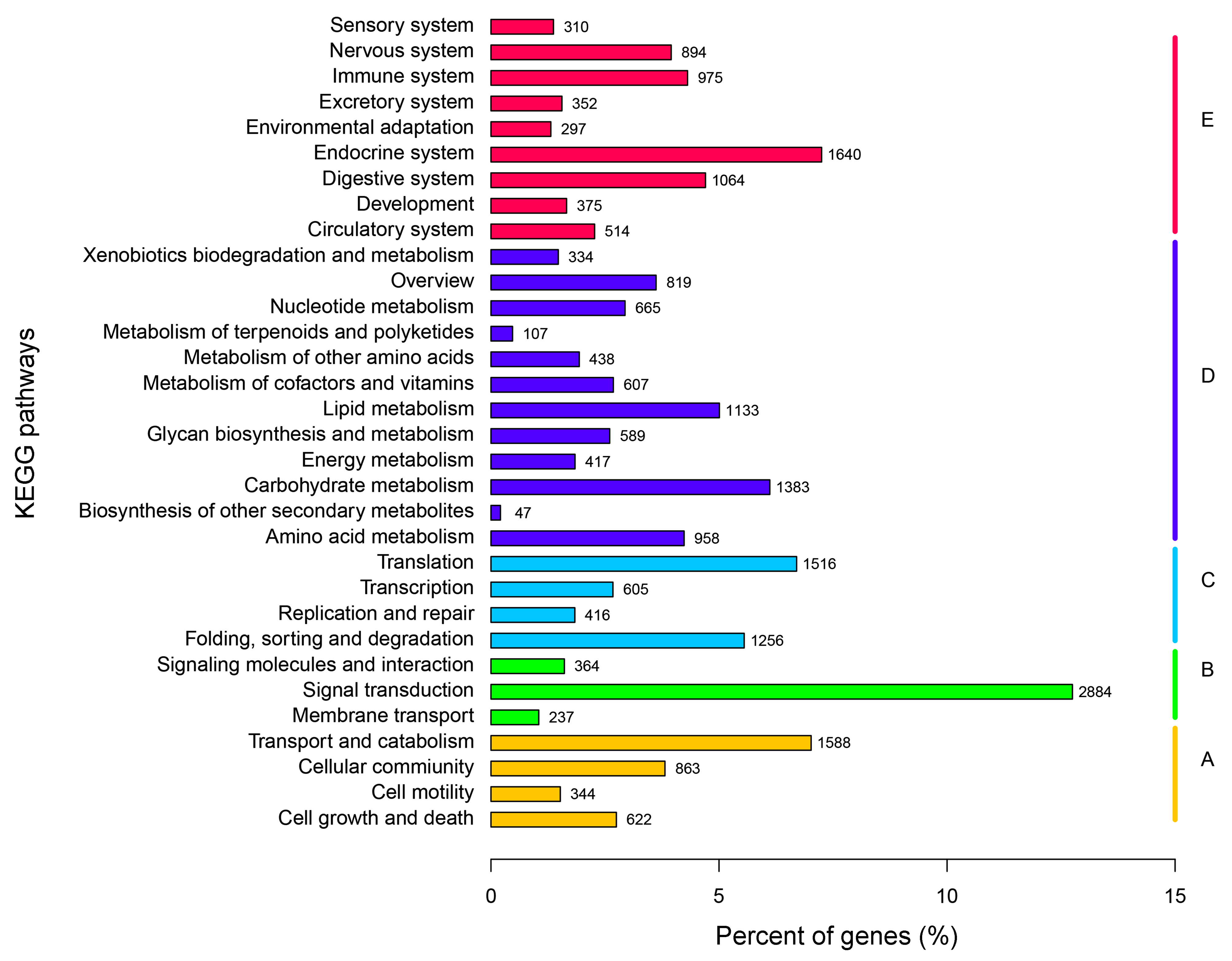

Supplement: Supplementary file 4 — Figure S3. KEGG pathway distributions of midgut unigenes. The genes according to KEGG metabolic pathway involved was divided into five branches: A, Cellular processes; B, Environmental information processing; C, Genetic information processing; D, Metabolism; E, Organismal systems (TIF 1056 kb) [file 12864_2019_5547_MOESM4_ESM.tif]

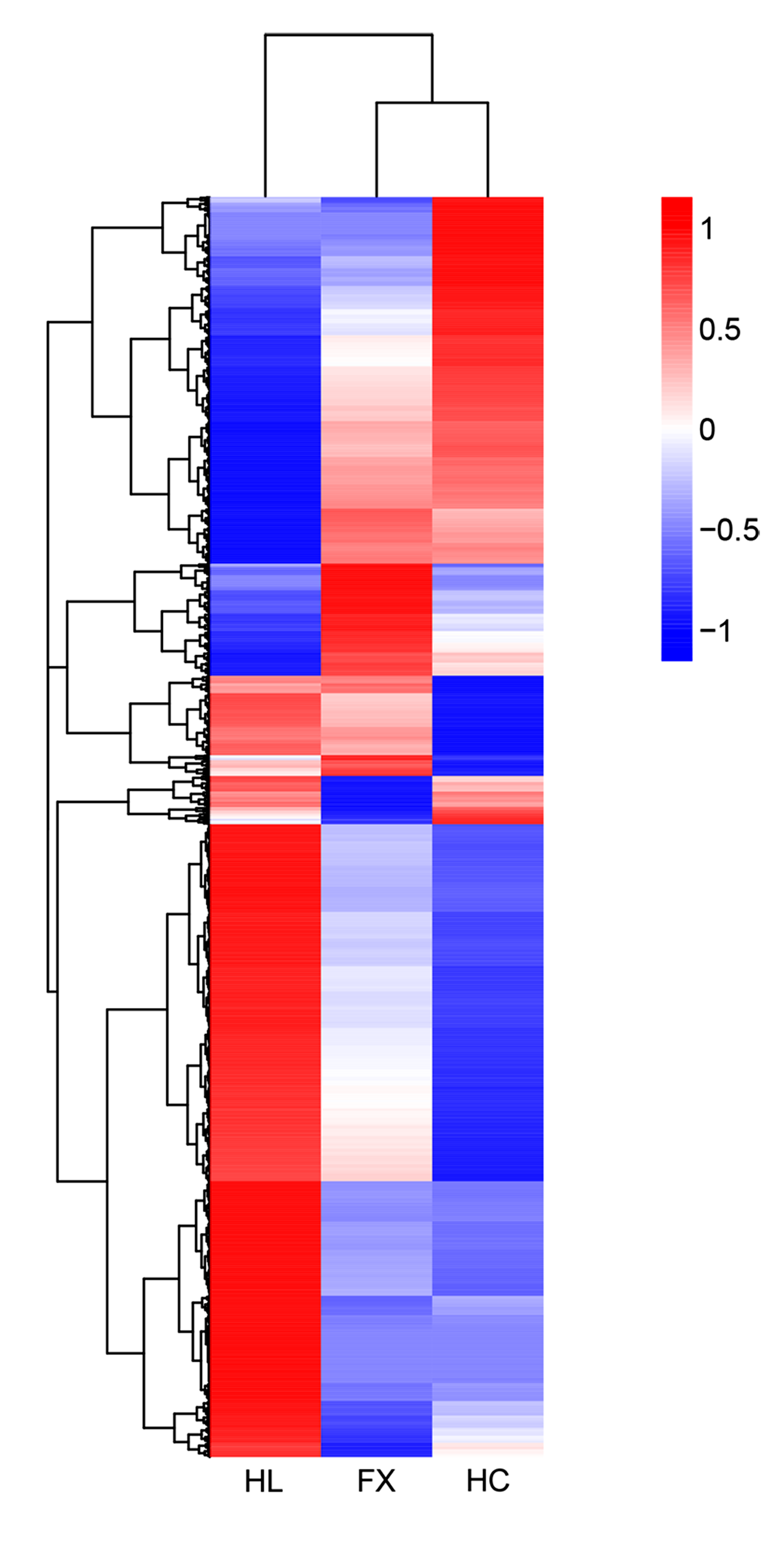

Supplement: Supplementary file 6 — Figure S4. Cluster analysis of differentially expressed genes. Different colors indicate different levels of gene expression: from red to blue, the log10(FPKM + 1) value ranges from large to small. FX: the population occurring in Fengxiang; HC: the population occurring in Hancheng; HL: the population occurring in the Helan Mountains (TIF 695 kb) [file 12864_2019_5547_MOESM6_ESM.tif]

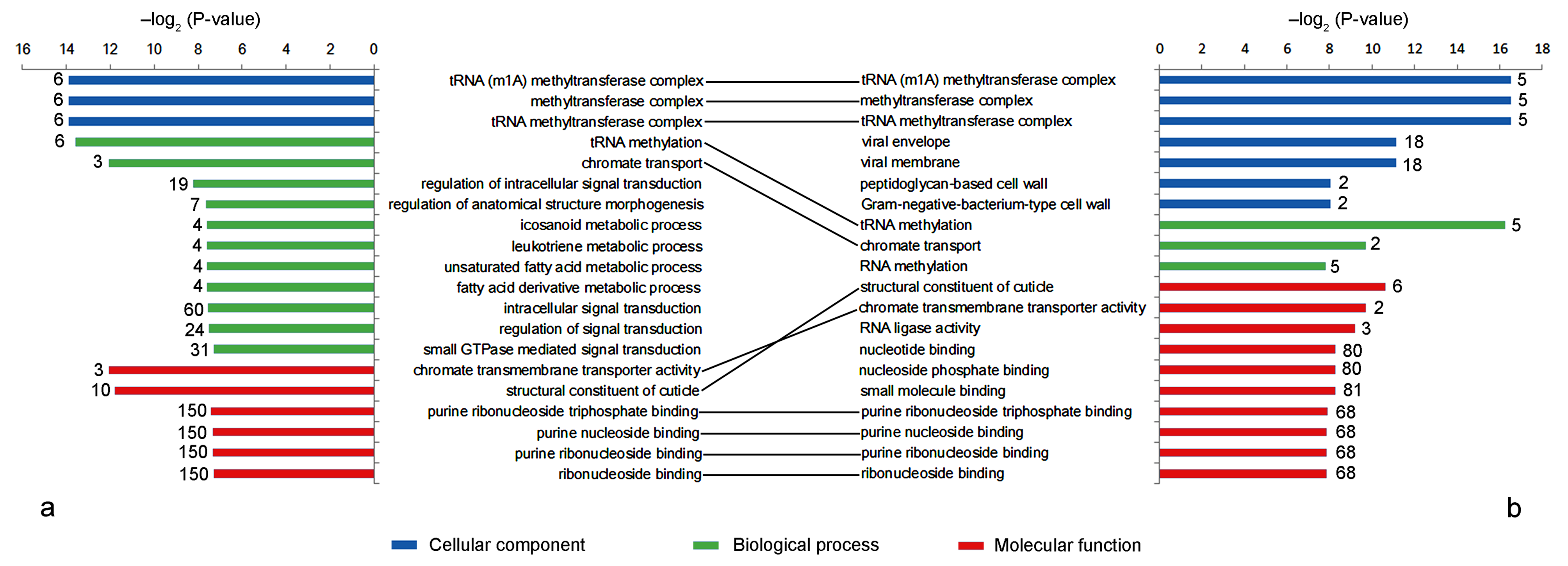

Supplement: Supplementary file 13 — Figure S5. GO enrichment of differentially expressed genes in (a) ‘HL vs HC’ and (b) ‘HL vs FX’. The 20 most enriched GO terms are shown together with their –log2(P-value) and number of genes (adjacent the bars). FX: the population occurring in Fengxiang; HC: the population occurring in Hancheng; HL: the population occurring in the Helan Mountains (TIF 1528 kb) [file 12864_2019_5547_MOESM13_ESM.tif]
